# Complications of Semirigid Ureteroscopy in Ureteric Stone Treatment

**DOI:** 10.7759/cureus.87896

**Published:** 2025-07-14

**Authors:** Jigardeep Singh, Vishwajeet Singh, Satyanarayan Sankhwar, Manoj Kumar, Udham Singh

**Affiliations:** 1 Urology, King George's Medical University, Lucknow, IND

**Keywords:** clavien-dindo, complications, satava, ureteroscopy, urolithiasis

## Abstract

Introduction

Ureteroscopy (URS) is a minimally invasive endoscopic technique commonly used to manage ureteric stones. This study prospectively reports and grades the complications associated with semirigid URS for ureteric stone removal and aims to identify factors contributing to these complications.

Methods

Prospective data were collected from 160 consecutive patients who underwent semirigid URS for ureteric stones at a single center between June 2021 and December 2022. Intraoperative complications were classified using Satava’s system, while postoperative complications were categorized according to the Modified Clavien-Dindo classification.

Results

The overall complication rate was 19.4%. Intraoperative complications occurred in 9.4% of cases, postoperative complications in 10.6%, and 0.6% experienced both. The most frequent intraoperative complication was failure to reach the stone (3.8%), while postoperative fever was the most common postoperative complication (4.4%). Larger stone size (p = 0.03), proximal stone location (p < 0.001), and presence of comorbidities (p = 0.012) were significantly associated with higher complication rates.

Conclusions

Semirigid URS is generally a safe and effective treatment for ureteric stones, especially in patients with favorable stone characteristics and comorbidity profiles. However, caution is advised when managing larger or proximally located stones and in patients with multiple comorbidities, as these factors increase the risk of complications. Future research should focus on multicenter studies with larger cohorts and longer follow-up periods to better understand late complications such as ureteric strictures. Additionally, investigating the impact of surgeon experience and patient demographics on complication rates could help optimize case selection and management strategies in URS.

## Introduction

Ureteroscopy (URS) is a well-established diagnostic and therapeutic approach in urological practice. Technological advancements and refinements in surgical techniques have expanded the scope of conditions treatable with URS, including renal or ureteric stones, urothelial carcinoma, and urethral strictures.

Minimally invasive surgical options for managing ureteral stones include shockwave lithotripsy (SWL), URS, percutaneous nephrolithotomy (PCNL), and laparoscopic or robotic-assisted stone surgery. URS is often the preferred method for ureteric stone management due to its high stone-free rate (SFR) and relatively low complication rates compared to SWL and PCNL [1,2].

URS achieves an overall SFR of 81-94%, which varies depending on the stone’s location within the ureter [3]. Specifically, the SFR is 81% for proximal ureter stones, 86% for mid-ureter stones, and 94% for distal ureteric stones.

Recent guidelines report a complication rate of 9-25% following URS [4]. These complications are generally classified as minor or major. Minor complications include fever, hematuria, and febrile UTIs, while major complications consist of ureteral perforation, avulsion, and strictures [5]. Notably, minor complications are underreported in the literature, as most studies focus primarily on major complications [6,7].

Postoperative urosepsis has been reported in approximately 5% of cases [8,9]. Significant risk factors for complications include prior ureteral perforations, positive preoperative urine cultures, patient comorbidities, and prolonged operative times [10,11]. Preventative strategies, such as administering prophylactic antibiotics, reducing stent dwell and operative times, identifying and treating UTIs, and carefully planning procedures for patients with a large stone burden or multiple comorbidities, can help minimize infectious complications following URS [12].

Currently, the European Association of Urology guidelines do not endorse a standardized classification system for URS-related complications. However, several scoring systems are available in the literature, including the Clavien-Dindo [13,14], Satava [1], and Post-Ureteroscopy Lesion Scale (PULS) [15] classifications. While Clavien-Dindo is the most widely used system for grading surgical complications in general, the Satava and PULS systems are more specific to URS.

The Clavien-Dindo classification was first applied to URS in 2012 by Mandal et al. to categorize and grade perioperative complications encountered during stone removal procedures. It stratifies complications occurring within three months post-surgery into five grades, with grades 1 and 2 considered “minor” and grades 3 to 5 classified as “major” [16].

The Satava classification groups complications into three grades: grade 1 includes those without clinical consequences, grade 2 involves complications requiring endoscopic intervention, and grade 3 requires open or laparoscopic surgery [1].

This study aims to determine the incidence and types of intraoperative and postoperative complications associated with semirigid URS for ureteric stones. It also seeks to identify key predictive factors - such as stone characteristics, patient comorbidities, and procedural variations - that may influence complication rates, with the ultimate goal of improving clinical decision-making and patient outcomes in URS.

## Materials and methods

Study design

This single-center prospective observational study was conducted in the Department of Urology at King George’s Medical University, Lucknow, India, from June 2021 to December 2022. Patients aged 18 years and above with ureteric stones who provided informed consent for ureteroscopic stone removal were included. Exclusion criteria were bilateral ureteric stones, anatomical abnormalities, or high-risk comorbidities that could potentially confound outcomes. Ethical clearance for the study was obtained from the institutional review board (IEC approval 2220/Ethics/2023).

Preoperative assessments included complete blood counts, serum creatinine, coagulation profile, and urine culture with sensitivity testing. A urine culture was considered sterile only in the absence of bacterial growth. Patients with abnormal laboratory results (e.g., elevated serum creatinine) underwent further evaluation before proceeding with URS. Radiologic assessments consisted of ultrasound of the kidney, ureter, and bladder (USG-KUB), X-ray KUB, and CT urography. Patients allergic to contrast or with renal insufficiency underwent non-contrast computed tomography.

Surgical procedure

All patients underwent semirigid URS under either regional or general anesthesia. Prophylactic intravenous broad-spectrum antibiotics were administered prior to surgery. The procedure began with cystoscopy and the insertion of a hydrophilic guidewire into the renal collecting system under fluoroscopic guidance. A 6/7.5 Fr semirigid ureteroscope (Richard Wolf GmbH, Knittlingen, Germany) was used. If the ureteroscope could not be advanced into the ureteric orifice, balloon dilatation was performed using a ureteral balloon dilator (Cook Medical, Bloomington, IN, USA).

After stone localization, fragmentation was performed using either a pneumatic lithotripter (Swiss LithoClast, EMS, Nyon, Switzerland) or a Holmium:YAG laser (Lumenis Pulse 100H, Boston Scientific, Marlborough, MA, USA), depending on stone size and location. Pneumatic lithotripsy was preferred for large, distal stones, while the holmium laser was used for smaller, proximal stones. Stone fragments were retrieved using tri-prong or bi-prong graspers. A 5/26 Fr DJ stent was placed at the end of the procedure in cases of impacted stones, mucosal injury, or residual fragments.

Postoperative

Antibiotic prophylaxis was continued during hospitalization. On the first postoperative day, all patients underwent blood count and serum creatinine testing. The urethral catheter was removed if there was no hematuria. Plain film KUB was performed on postoperative day 1 to assess stone clearance and verify DJ stent position. Patients were discharged unless further management was required.

Fever was defined as a body temperature ≥100°F (37.7°C). Transient hematuria was defined as hematuria lasting for at least six hours but resolving spontaneously within 48 hours. Persistent hematuria refers to hematuria lasting beyond 48 hours. Follow-up imaging, including plain film and USG-KUB, was performed two weeks post-surgery, and the DJ stent was subsequently removed under local anesthesia.

Complications were categorized as intraoperative or postoperative. Intraoperative complications were graded using the modified Satava classification system [1], while postoperative complications were classified according to the modified Clavien-Dindo system [13,14].

Statistical analysis

Categorical variables were expressed as frequencies and percentages, and continuous variables were presented as means with SDs. Statistical analyses were performed using the chi-square test for categorical variables and the t-test for continuous variables, with adjustments made for multiple comparisons. A p-value <0.05 was considered statistically significant. Data entry was performed using Microsoft Excel (Microsoft Corporation, Redmond, WA, USA), and statistical analysis was conducted using IBM SPSS Statistics for Windows, Version 23.0 (Released 2015; IBM Corp., Armonk, NY, USA).

## Results

Out of 160 patients, 31 (19.4%) experienced complications. Based on the location relative to the sacroiliac joint, stones were categorized as upper ureteric (above the joint) or lower ureteric (below the joint), with 70 patients (43.8%) and 90 patients (56.2%) in each group, respectively. For statistical analysis, stone size was classified into two groups: ≤10 mm and >10 mm, with 69 patients (43.1%) and 91 patients (56.9%) in each category. In our study, 70% of patients presented with non-impacted stones, while 30% had impacted stones. All patients had sterile preoperative urine cultures and sensitivity tests. A total of 24 patients (15%) had comorbid conditions, with hypertension being the most common, occurring in nine patients (5.6%). The demographic characteristics of the study population are summarized in Table 1.

**Table 1:** Demographic details of the study cohort

Variable	Frequency (n)	Percentage (%)
Age (years) (mean ± SD)	-	37 ± 11
Age group		
18-35 years	79	49.4
36-50 years	58	36.2
51-65 years	22	13.8
>65 years	1	0.6
Total	160	100
Gender		
Male	107	66.9
Female	53	33.1
Total	160	100
Comorbidity		
Chronic kidney disease	5	3.1
Hypertension	9	5.6
Type 2 diabetes mellitus	6	3.8
Hypertension and type 2 diabetes mellitus	1	0.6
Hypothyroidism	3	1.9
None	136	85
Total	160	100

Table 2 provides a comparative overview of patients with and without complications, along with descriptive data. Of the total complications observed, 15 patients (9.4%) experienced isolated intraoperative complications, 17 patients (10.6%) had isolated postoperative complications, and one patient (0.6%) experienced both intraoperative and postoperative complications.

**Table 2:** Comparison of descriptive characteristics between patients with and without complications

Variables		Complications				p-value
		Yes		No		
		N	%	N	%	
Sex	Male	19	17.80%	88	82.20%	0.462
	Female	12	22.60%	41	77.40%	
	Total	31	19.40%	129	80.60%	
Laterality	Bilateral	1	100.00%	0	0.00%	0.122
	Left	15	19.20%	63	80.80%	
	Right	15	18.50%	66	81.50%	
	Total	31	19.40%	129	80.60%	
Stone size	≤10 mm	8	11.60%	61	88.40%	0.03
	>10 mm	23	25.30%	68	74.70%	
	Total	31	19.40%	129	80.60%	
Stone location	Lower ureter	6	6.70%	84	93.30%	<0.001
	Upper ureter	25	35.70%	45	64.30%	
	Total	31	19.40%	129	80.60%	
Lithotripter type	HO:YAG	23	18.40%	102	81.60%	0.977
	Pneumatic	8	22.80%	27	77.20%	
	Total	31	19.40%	129	80.60%	
Impacted	Yes	11	22.90%	37	77.10%	0.458
	No	20	17.90%	92	82.10%	
	Total	31	19.40%	129	80.60%	
Comorbidity	Chronic kidney disease	3	60.00%	2	40.00%	0.012
	Hypertension	4	44.40%	5	55.60%	
	Hypertension and type 2 diabetes mellitus	1	100.00%	0	0.00%	
	Hypothyroidism	0	0.00%	3	100.00%	
	None	22	16.20%	114	83.80%	
	Type 2 diabetes mellitus	1	16.70%	5	83.30%	
	Total	31	19.40%	129	80.60%	

**Figure 1:**
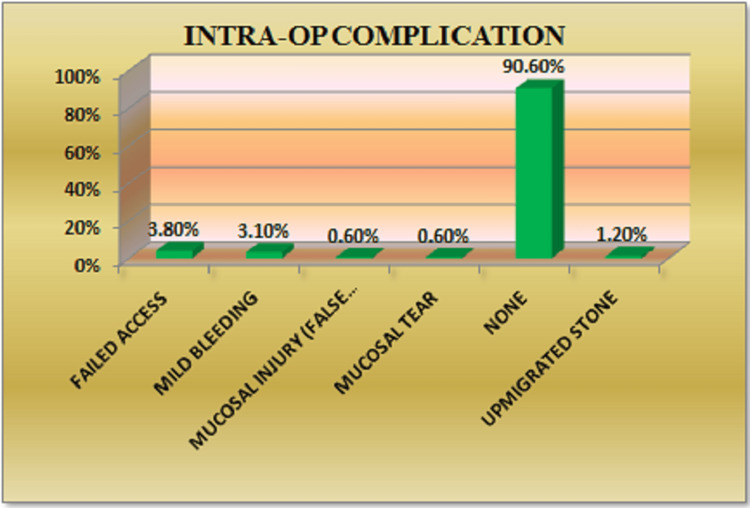
Distribution of patients according to intraoperative complications

The most common intraoperative complication observed was the inability to reach the stone (3.8%), followed by mild mucosal bleeding (3.1%) (Figure 1). Intraoperative complications were categorized using the Modified Satava grading system. Among these, Grade I complications were the most frequent, accounting for 40%, while Grade III complications were the least common, observed in 6.7% of cases. Table 3 and Table 4 present the descriptive data related to intraoperative complications. 

**Table 3:** Descriptive information on intraoperative complications

Intraoperative complication	Frequency (n)	Percentage (%)
Failed access (inability to reach the stone)	6	3.8
Mild bleeding	5	3.1
Mucosal injury (false passage/route)	1	0.6
Mucosal tear	1	0.6
Upmigrated stone	2	1.2
No complication	145	90.6
Total	160	100

**Table 4:** Intraoperative complications classified according to the modified Satava classification system

Modified Satava classification	Frequency (n)	Percentage (%)
Grade I	6	40
Grade IIA	3	20
Grade IIB	5	33.3
Grade III	1	6.7
Total	15	100

**Figure 2:**
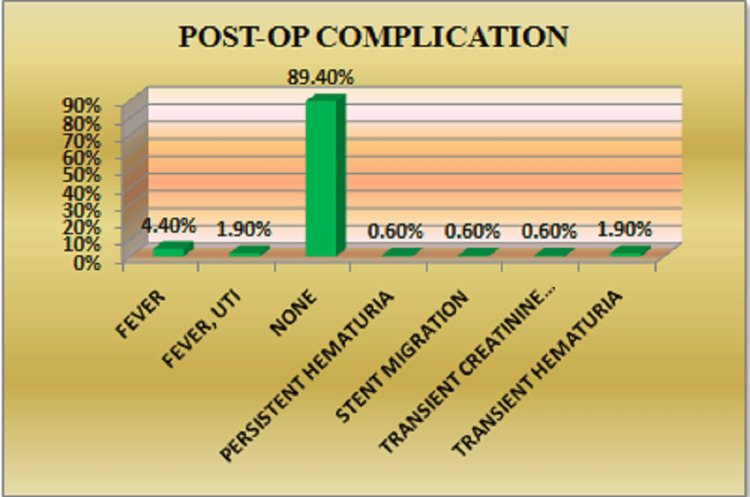
Distribution of patients according to postoperative complications

In this study, 17 patients (10.6%) experienced postoperative complications. The most common was fever (4.4%), followed by febrile UTI and transient hematuria, each occurring in 1.9% of patients (Figure 2). Postoperative complications were categorized using the Modified Clavien-Dindo classification system. Grade I complications were the most prevalent (70.6%), followed by Grade II (17.6%), Grade IIIB (5.9%), and Grade IVB (5.9%). No Grade IIIA, IVA, or V complications were observed.

Table 5 and Table 6 provide descriptive details and Clavien-Dindo classification of postoperative complications, respectively. Patients who experienced complications had longer hospital stays (4 ± 1 days) and longer operative times (60 ± 16 minutes).

**Table 5:** Descriptive information on postoperative complications

Postoperative complication	Frequency (n)	Percentage (%)
Fever	7	4.4
Febrile UTI	3	1.9
Persistent hematuria	1	0.6
Stent migration	1	0.6
Transient creatinine elevation	1	0.6
Transient hematuria	3	1.9
Urosepsis	1	0.6
No complications	143	89.4
Total	160	100

**Table 6:** Postoperative complications classified according to the modified Clavien-Dindo classification system

Clavien-Dindo classification	Frequency (n)	Percentage (%)
Grade I	12	70.6
Grade II	3	17.6
Grade IIIB	1	5.9
Grade IVB	1	5.9
Total	17	100

## Discussion

In this prospective observational study, complications among 160 patients who underwent semirigid URS for ureteric stones were analyzed. Complications were observed in 31 patients (19.4%). Of these, 15 patients (9.4%) experienced intraoperative complications, 17 patients (10.6%) experienced postoperative complications, and one patient (0.6%) experienced both intraoperative and postoperative complications. Overall, the most common complication was fever (4.4%), followed by failure to access the stone (3.8%).

According to the CROES URS global study [17], the overall complication rate was 7.4%, which is lower than the rate observed in our study. This discrepancy may be attributed to the fact that many procedures in our cohort were performed by trainee residents or junior faculty, potentially contributing to variability in outcomes due to differences in experience. Additional contributing factors may include the prospective nature of our study, in contrast to retrospective designs, as well as the relatively small sample size. Nevertheless, a review of the literature shows that complication rates for URS in ureteric stone management range from 9% to 25%, aligning with our findings [4].

In this study, 24 patients (15%) had comorbidities. Among them, nine patients (37.5%) experienced complications. In contrast, among the 136 patients without comorbidities, 22 (16.1%) developed complications, a trend consistent with the findings of Somani et al. [17].

We also assessed the relationship between stone size and complication rates. A statistically significant association was observed (p = 0.03). Specifically, eight of 69 patients (11.6%) with stones ≤10 mm experienced complications, compared to 23 of 91 patients (25.3%) with stones >10 mm.

Complications were also analyzed based on stone location. A statistically significant association was found (p < 0.01). Among 70 patients with upper ureteric stones, 25 (35.7%) experienced complications, compared to six of 90 patients (6.7%) with lower ureteric stones. This trend is consistent with the findings of Mandal et al. [16], who conducted a prospective study on URS-related complications.

The relationship between stone impaction and complication rates was also examined. No statistically significant association was found (p = 0.458). Complications occurred in 11 of 48 patients (22.9%) with impacted stones and in 20 of 112 patients (17.8%) with non-impacted stones.

We further evaluated complication rates based on the type of lithotripter used. Among 125 patients who underwent Ho:YAG laser lithotripsy, 23 (18.4%) experienced complications, compared to 8 of 35 patients (22.8%) who underwent pneumatic lithotripsy. These findings are consistent with those of Maghsoudi et al. [18], who found no significant differences in complication rates - such as perforation, mucosal injury, and bleeding - between the two lithotripter types.

Thus, complications following URS can be minimized by identifying and treating UTIs preoperatively and by planning carefully in patients with a large stone burden or significant comorbidities.

Meaning and limitations of the study

The prospective design of this study supports reliable data collection and reduces recall bias, thereby improving the accuracy of reported complication rates. The use of both the Satava and Clavien-Dindo classification systems allowed for comprehensive grading of intraoperative and postoperative complications, including minor events such as mucosal injuries, which are often underreported. Furthermore, the identification of predictive factors - such as stone size, location, and patient comorbidities - offers valuable insights to guide clinical decision-making in URS.

However, the study has several limitations. The relatively short follow-up period may not capture late-onset complications, such as ureteric strictures, which can develop months after the procedure. Additionally, being a single-center study limits generalizability, as patient demographics and surgical expertise may vary across institutions. Lastly, many of the procedures were performed by trainee residents or junior faculty, potentially introducing variability in complication rates due to differences in experience. Future studies with multicenter designs and extended follow-up periods are warranted to validate and expand upon these findings.

## Conclusions

Based on the observed complication rates, semirigid URS is generally a safe and effective treatment for ureteric stones, especially when patient selection considers stone characteristics and comorbidity profiles. However, special caution is advised in cases involving larger, proximal ureteric stones or patients with multiple comorbidities, as these factors are associated with higher complication rates. Our findings indicate that stone size, location, and comorbidities are significantly associated with complication risk. Larger stones, proximal location, and the presence of comorbidities were linked to higher complication rates. Other factors - such as age, gender, stone laterality, and lithotripter type - were not significantly associated with complications. Future research should focus on multicenter studies with larger cohorts and longer follow-up periods to capture delayed complications such as ureteric strictures. Further investigation into the effects of surgeon experience and patient demographic variability may help refine case selection and optimize management strategies for URS.
